# Effects of Sex, Age and Height on Symphysis–Ischial Spine Distance Measured on a Pelvic CT

**DOI:** 10.3390/jcm11092395

**Published:** 2022-04-24

**Authors:** Daniel Sánchez García, Alejandra Aguado del Hoyo, María Sánchez Pérez, Santiago García-Tizón Larroca, Yolanda Ruiz Martín, Isabel Gordillo Gutiérrez, Coral Bravo Arribas, Melchor Alvarez-Mon, Miguel A. Ortega, Juan De Leon-Luis

**Affiliations:** 1 Department of Radiology, University Hospital Gregorio Marañón, 28009 Madrid, Spain; dsgarcia@salud.madrid.org (D.S.G.); alejandra.aguado@salud.madrid.org (A.A.d.H.); maria.sanchez.perez@salud.madrid.org (M.S.P.); yolandajose.ruiz@salud.madrid.org (Y.R.M.); isabel.gordillo@salud.madrid.org (I.G.G.); 2 Group of Pathophysiology in Women, Pregnancy, Labor, and Puerperium, Health Research Institute Gregorio Marañón, 28040 Madrid, Spain; gineteca@gmail.com (S.G.-T.L.); jaleon@ucm.es (J.D.L.-L.); 3 Maternal and Infant Research Investigation Unit, Alonso Family Foundation (UDIMIFFA), 28009 Madrid, Spain; 4 Department of Public and Maternal and Child Health, School of Medicine, Complutense University of Madrid, 28040 Madrid, Spain; 5 Department of Obstetrics and Gynecology, University Hospital Gregorio Marañón, 28009 Madrid, Spain; 6 Department of Medicine and Medical Specialities, Faculty of Medicine and Health Sciences, University of Alcalá, 28801 Alcala de Henares, Spain; mademons@gmail.com (M.A.-M.); miguel.angel.ortega92@gmail.com (M.A.O.); 7 Ramón y Cajal Institute of Sanitary Research (IRYCIS), 28034 Madrid, Spain; 8 Immune System Diseases-Rheumatology, Oncology Service an Internal Medicine (CIBEREHD), University Hospital Príncipe de Asturias, 28806 Alcala de Henares, Spain

**Keywords:** symphysis–ischial spine distance, pelvic CT images, reproducibility

## Abstract

Objective: To examine the influence of age, sex and height on the symphysis–ischial spine distance (SID) measured on pelvic Computed tomography (CT)images in subjects of reproductive age, and to determine the interobserver reproducibility. This measurement (SID) is of great importance because the use of intrapartum ultrasound is based on the assumption of a specific value (30 mm) of such a measurement. Methods: This was a cross-sectional descriptive study in which SID was measured in subjects aged 20 to 44 years who had been scheduled for pelvic CT at our centre from January 2018 to May 2021 for different reasons. Radiographic measurements of the pelvis were obtained through the multiplanar reconstruction of the CT image. The images obtained from all of the participants were independently assessed by three senior radiologists, and the SID measurements made by each one were blinded from those of the remaining observers. Correlations between the SID and patient age, height and sex were analyzed by univariate and multivariate linear regression. Results: The mean SID for 87 of the enrolled participants (45 women, 42 men) was 28.2 ± 6.25 mm. Among the observers, the mean difference in this distance was 1 to 2 mm, and was scarcely related to measurement size, with agreement being greater than 70%. The mean SID was significantly related to sex and height (SID = −24.9 − 6.51 × sex (0 or 1) + 0.34 × height (cm); *p* = 0.01; sex equals 1 for a man and 0 for a woman), such that it was a mean of 2.5 mm greater in women than men (29.50 mm vs. 26.99 mm). Conclusion: Measurements of SID on CT images show good interobserver reproducibility, and are related to sex and height.

## 1. Introduction

Intrapartum ultrasound accompanied by transvaginal digital examination is being used increasingly often [1,2,3,4,5,6,7,8,9,10,11,12,13] to determine foetal head station in the second stage of labour, and thus to establish the chances of a successful vaginal delivery. This approach, although poorly reproducible, is still considered the gold standard procedure [14,15,16,17,18].

One of the landmarks assessed by intrapartum ultrasound is foetal head station, and the head is said to be engaged in the pelvis at 0 station or Hodge plane III. When in this position, the chances of a successful vaginal delivery are high and, if necessary, an instrumental delivery is considered low risk. The 0 station matches the anatomical transverse plane of the mother’s ischial spines. Therefore, it is of great interest to identify this bony structure in the vaginal or ultrasound patient assessment. Unfortunately, however, ultrasound does not allow for the proper visualization of this region [19], and the distance between the infrapubic plane and ischial spine plane (symphysis–ischial spine distance, SID) is normally assumed to be 30 mm [3], according to different authors [20,21,22].

As a complement to ultrasound, computed tomography offers greater reliability in the quantification of measurements, especially for bone structures [23]. Furthermore, owing to the possibility of multiplanar and volumetric reconstructions [24,25] with this technique, it is widely used for bone biometry measurements, as described by Friedman et al. [26], Mullaji et al. [27] and Cai et al. [28], among others.

The shape of the female pelvis varies widely, such that several types have been described: gynecoid (45%), android (30%), anthropoid (20%) and platypelloid (5%). In men, the pelvis is mainly of the android type [26,27,28]. In order to determine the pelvis shape type an exhaustive examination is needed, involving numerous measurements [24,25]. The ideal pelvic shape for a vaginal delivery is the gynecoid type [24,25]. Hence, by comparing pelvic measurements in young adults, we could explore the effect of sex on these measurements. While several studies have provided SID measurements, few have examined variations which are attributable to the effects of different anthropometric and demographic factors such as age, sex and height.

The main aims of this study were to calculate the mean SID for a population of young adults who were subjected to a pelvic CT for various indications, in order to examine the effects on these measurements of age, sex and height, and to establish the interobserver reproducibility of the measures made.

## 2. Materials and Methods

This was a descriptive, observational, cross-sectional study. Symphysis–ischial spine distance (SID) measurements were made on abdominopelvic CT images acquired from individuals aged 20 to 44 years at our centre over the period of January 2018 to May 2021. Among the inclusion criteria were: an adequate quality of multiplanar CT reconstructions, the absence of scoliosis or the metastatic involvement of the pelvic bone, and a lack of pelvic fractures or sequellae of pelvic fractures. Based on the cohort of patients fulfilling our inclusion criteria, sampling was conducted through a randomized analysis stratified by sex. In each participant, we collected clinical variables including age (years), sex and height (cm) from the data recorded in the pre-procedural exam at the time of the CT scan. The study protocol received approval from the Ethics Committee for Medical Research of our centre (Code: OBS 29112021).

### 2.1. CT Measurements

The CT scans were performed using one of three devices: Ingenuity CT (Philips, Eindhoven, The Netherlands), Brilliance 64 (Philips, Eindhoven, The Netherlands) and Brilliance 16 (Philips, Eindhoven, The Netherlands), which have 128, 64 and 16 channels, respectively. The field of view includes the entire abdomen and pelvis. The voltage used in all of the studies was 120 kV. The slice thickness was 2 mm in all of the devices. The analysis of the images was performed with the bone window. Radiographic measurements of the pelvis were obtained from the multiplanar reconstructions (MPR) of the CT scans, as opposed to the volumetric reconstructions described by other authors [21].

**Figure 1:**
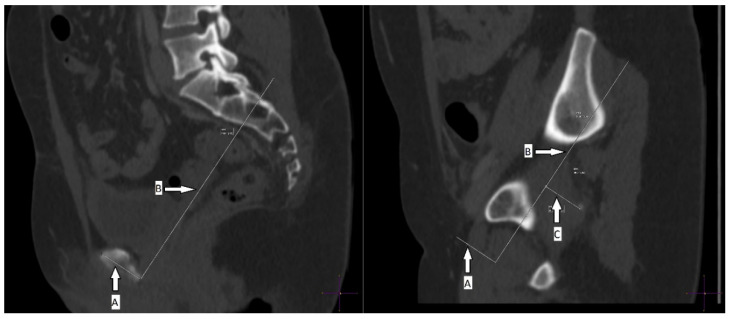
Lines A and B are drawn on the sagittal plane that crosses the pubic symphysis (**left**). Lines A, B and C are drawn on the plane that crosses the top of the left ischial spine. Line C is the SID distance (**right**). symphysis–ischial spine distance (SID).

On a sagittal plane crossing the pubic symphysis, a line is drawn following the axis of the pubic symphysis to its lower margin (line A). From this point, a second line is drawn in a posterior direction perpendicular to line A in the same plane (line B). The two lines drawn on the pubic symphysis plane are then transferred to the sagittal plane that crosses the top of the left ischial spine, as is normally performed for orthopaedic measurements [28]. In order to obtain the SID (in mm), a perpendicular line is drawn joining line B to the top of the ischial spine (line C) (Figure 1).

In order to assess the reproducibility, SID measurements were made on CT scans for each participant independently by three senior radiologists (A, B and C). The measurements made by the most experienced radiologist (A) served as a reference. The remaining observers were blind to the observations made by each one. The outcome variable was the mean and standard deviation (SD) of the SID values obtained by each observer.

### 2.2. Statistical Analysis

All of the data acquired were entered into a Microsoft Office Excel database, version 15.0.4420.1017 (Microsoft, Redmond, WA, USA), for subsequent analysis. The quantitative variables are expressed as the mean and standard deviation (SD), and the categorical variables are expressed as a number and percentage (95% CI). The Kolmogorov–Smirnov test was used to establish the normality of the data. Student’s *t*-test was used to compare the age and height data and SID measurements according to sex. The statistical analysis was performed using SPSS Version 26.0 (IBM Corp., Armonk, NY, USA) with its default settings. Significance was set at *p* < 0.05.

The agreement between the radiologists’ measurements was assessed through Lin’s concordance correlation coefficient and Bland–Altman analysis [29,30,31]. In order to assess the interobserver reproducibility, we calculated intraclass correlation coefficients (ICC), using the cutoffs >0.7, 0.5 to 0.7, and <0.5 to indicate good, intermediate and poor correlation, respectively.

For our analysis of factors related to variations in SID, we used as reference the measurements made by observer A (the most experienced observer). Through univariate and multivariate linear regression analysis, we described the relationship of the mean SID with age, sex and height, including the multivariate model’s clinically relevant variables or those showing a *p* < 0.2.

The study protocol received approval from the Ethics Committee for Medical Research of our centre (Code: OBS 29112021).

## 3. Results

Over the study period, acceptable quality MPRs of abdominopelvic CT images were obtained in 87 patients fulfilling the inclusion criteria.

Table 1 describes the clinical characteristics of the participants, along with the mean SID measurements obtained for the whole population and separately for the men and women.

**Figure 2:**
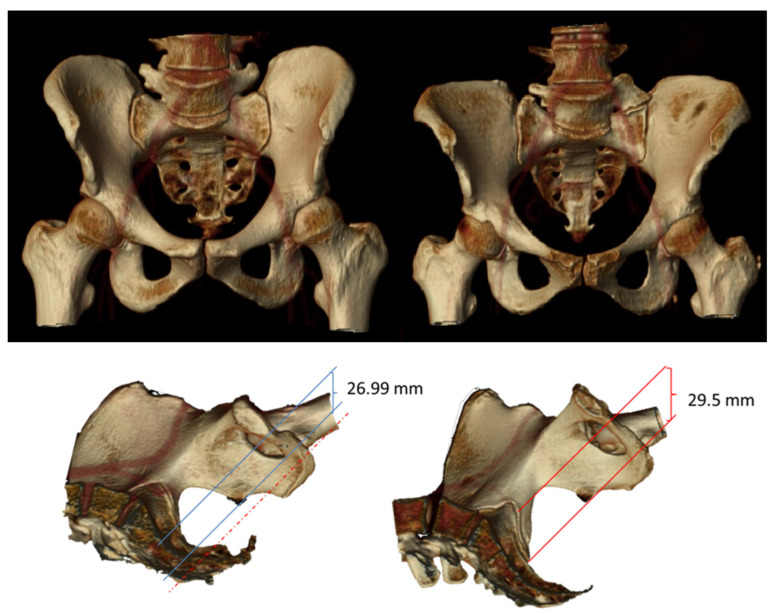
Three-dimensional reconstructions of a male and female pelvis in the coronal (**top**) and sagittal (**bottom**) planes through the pubic symphysis. Note the different morphologies of the male and female pelvis. Especially noticeable in the coronal reconstruction is the difference in the pelvic bone. The sagittal reconstruction shows the mean symphysis–ischial spine distance (SID), as measured between the planes of the pubic symphysis and the ischial spine in men and women. Note its higher value in women (29.50 mm vs. 26.99). The dotted line in the left image marks on the male pelvis the mean SID obtained in the women.

Figure 2 shows the 3D reconstruction of a male (left) and female (right) pelvis in the coronal (frontal) and sagittal (anteroposterior) planes, indicating the SID means obtained for men and women.

**Figure 3:**
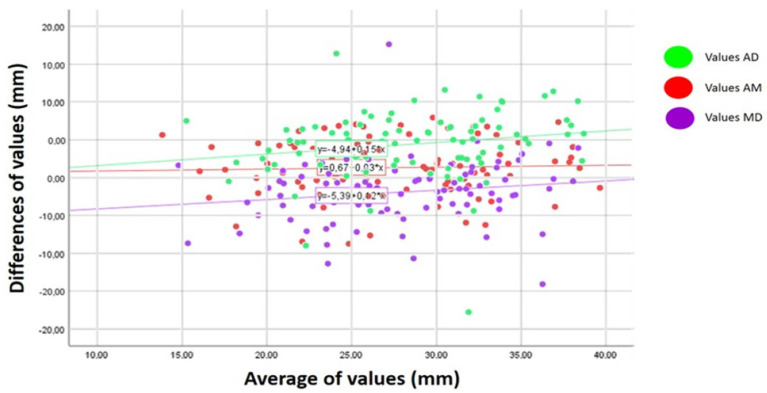
Bland–Altman plot of the mean differences in the mean symphysis–ischial spine distance (SID) values obtained by the observers (A–B, A–C and B–C). The regression lines indicate the trends shown by these differences.

The intraclass correlation coefficients (ICC) and their 95% confidence intervals (CI) obtained for the different pairs of observers are provided in Table 2. Figure 3 shows the mean differences in SID among the three observers.

The variations in the SID values were determined by age, sex and height. In Table 3, we describe the results of the univariate linear regression analysis of the SID values according to age, sex and height, and those of the multivariate analysis which did not include age.

Once an effect of age had been ruled out, our multivariate regression analysis yielded the following equation to calculate symphysis–ischial spine distance (SID) (mm):

SID=−24.9−6.51×sex*+0.34× height in cm (p < 0.01)

* where sex equals 1 for a man and 0 for a woman.

Applying this equation, for example, to a Spanish woman of height 163 cm (as the mean provided by the OCDE), the SID predicted is 30.5 cm, and for a Danish woman of 168 cm (as the mean provided by the OCDE), the SID would be estimated at 32.2 cm.

## 4. Discussion

Our study provides mean SID values for a study population of 87 subjects (45 women, 42 men) of reproductive age, as measured on an abdominopelvic CT scan. The results were a mean SID of 29.50 ± 5.67 mm for women and 26.99 ± 6.64 for men, albeit that this was not significantly different. Despite the lack of significance, the mean values were up to 2.5 cm higher in women than men. Our inter-observer reproducibility study indicated very good agreement between the measurements (ICC ≥ 0.7). Through multivariate linear regression, we also determined that sex and height had an independent effect on SID.

The literature studies that provided SID measurements have not included an analysis by age and sex. Compared to the SID values detected here, Arthuis et al. reported slightly lower values (26.7 ± 5.8 mm) for 458 women in their third pregnancy term in France, and Armbrust et al. recorded higher values (32.35 ± 4.46 mm) in 23 non-pregnant women in Germany. These discrepancies could be explained by height differences, as observed here. Both of these research groups described a significant direct relationship between the mean SID and height, with correlation coefficients of 0.09 obtained in the study by Arthuis et al., and of 0.5 in the study by Armbrust et al.

In our interobserver reproducibility study, we detected greater agreement between observers A and B (ICC 0.79) than between A and C or B and C (ICC 0.70). Among the published reports, only Arthuis et al. compared the results of two observers for 30 patients, and observed a similar ICC for SID measurements to that found here of 0.83 (95% CI 0.73–0.92). As in our study, these authors noted no clear trend in the difference between the observers. Despite the level of agreement, it could be that some differences between observers reflect their learning curves. Thus, the lower agreement observed for our observers A and B with C could reflect the known lesser experience of Radiologist C.

Our study describes the influence of age, sex and height on mean SID for a given cohort, and provides an equation to calculate the expected SID according to the latter two factors. No correlation was detected between the mean SID and subject age in the univariate or multivariate analysis. It was especially appreciable in the multivariate analysis that the mean SID was significantly higher in women than men, and that both values were directly related to height.

As in the case of SID, radiological biometries are often affected by height and sex, such as those described for the spleen [32], acetabular angle [33], several cardiovascular magnetic resonance measurements [34], and measurements of the thoracic aorta [35], etc.

Whilst the radiological classification of female pelvis type currently has scarce clinical applications [36], our finding of differences in SID between men and women suggests that a pelvis with more android characteristics could have a similar SID to that observed in men, along with other measurements (e.g., pelvic bone diameter), and these differences could have implications for the success of a vaginal delivery.

Among the strengths of our study, we should mention that it is the first to examine the impacts of sex on SID, and thus to consider its relationship with female pelvis shape as an essential biometry for the assessment of the progression of a vaginal delivery [4,20,21]. Just as others have examined the importance of height, we were able to determine the influence of sex on SID in relation to height. We were also able to show that SID is reproducible when measured by three observers with different levels of experience.

Among the limitations of this study are the facts that the participants were not gestating mothers and its sample size was small. In addition, obstetric characteristics such as parity and their possible effects on SID were not determined. It is, nevertheless, difficult to obtain a large sample size when exposing pregnant women to ionizing radiation. Our findings do, however, provide direction for future studies designed to examine the impacts of factors such as pelvis type or parity on pelvimetry.

## 5. Conclusions

The results of this study reveal a significant impact of height on SID measured on a pelvic CT; this distance is longer in women. The value of SID in our sample of women was in line with the clinical standard of approximately 30 mm used for intrapartum ultrasound. Thus, it seems that this form of measurement serves to more objectively estimate the height of foetal presentation during labour than transvaginal examination. The estimation of SID in a pelvic CT was found to be reproducible, showing little interobserver variation.

## Figures and Tables

**Table 1:** Clinical characteristics and symphysis–ischial spine distance (SID) measurements made for the whole population, and by sex or observer. The data are provided as the mean and standard deviation (SD); * *p* < 0.05.

Variables	Whole Population (*n* = 87)	Men (*n* = 42)	Women (*n* = 45)	*p*
Age (years)	31.98 ± 6.20	31.98 ± 6.37	31.81 ± 6.07	0.81
Height (cm)	169.55 ± 10.53	178 ± 7.28	162 ± 7.33	* 0.001
SID (mm)				
Observer A	28.20 ± 6.25	26.99 ± 6.64	29.50 ± 5.67	0.07
Observer B	26.83 ± 6.11	25.96 ± 5.91	27.59 ± 6.30	0.36
Observer C	28.79 ± 5.48	27.94 ± 5.49	29.65 ± 5.46	0.23

**Table 2:** Correlation between the mean symphysis–ischial spine distance (SID) measurements made by the three observers (A, B and C) and the 95% confidence intervals (CI).

	Intraclass Correlation Coefficient	95% CI
Observer A–B	0.79	0.68–0.87
Observer A–C	0.70	0.58–0.79
Observer B–C	0.70	0.52–0.81

**Table 3:** Results of the univariate linear regression analysis; * *p* < 0.05. confidence intervals (CI).

Univariate	Coefficient	95% CI	*p*
Age	−0.092	(−0.31 to 0.12)	0.4
Height	0.109	(−0.059 to 0.276)	0.199
Sex	−2.417	(−5.047 to 0.214)	0.07
Multivariate			
Height	0.34	(−0.11 to 0.56)	* 0.005
Sex	−6.51	(−11.30 to−1.72)	* 0.009

## Data Availability

The datasets used and/or analyzed during the present study are available from the corresponding author on reasonable request.
